# Giant intravesical prostatic protrusion mimicking bladder carcinoma: Navigating diagnostic and management challenges

**DOI:** 10.1016/j.ijscr.2024.109590

**Published:** 2024-03-27

**Authors:** Bartholomeo Nicholaus Ngowi, Mathias Kimolo, Jasper Mbwambo, Frank Bright, Alex Mremi, Orgeness Jasper Mbwambo

**Affiliations:** a Faculty of medicine, Kilimanjaro Christian medical university college, P. O. Box 2240, Moshi, Tanzania; b Department of urology, Kilimanjaro Christian Medical Centre, P. O. Box 3010, Moshi, Tanzania; c Department of pathology, Kilimanjaro Christian Medical Centre, P. O. Box 3010, Moshi, Tanzania

**Keywords:** IPP, BPH, Bladder carcinoma

## Abstract

**Introduction and importance:**

Benign prostate hyperplasia is common condition among elderly men, but giant intravesical prostatic protrusion is rare and may be confused with bladder carcinoma.

**Case presentation:**

We report an unusual case of giant intravesical prostatic protrusion mimicking bladder carcinoma. A diagnosis of giant intravesical prostatic protrusion was confirmed with the assistance of cystoscopy and patient was managed by transvesical simple open prostatectomy where he had uneventfully recovery.

**Clinical discussion:**

Both bladder carcinoma and benign prostate hyperplasia are more prevalent in elderly men and they all present with lower urinary tract symptoms. Ultrasound and computer tomography may all suggest bladder carcinoma. The two conditions are treated differently, and therefore having correct diagnosis is mandatory. Cystoscopy is an important investigation that can act as a tiebreaker in differentiating giant intravesical prostatic protrusion from bladder carcinoma. Transvesical simple open prostatectomy is the preferred surgical approach with good postoperative outcome.

**Conclusion:**

This case report reminds urology surgeons on the possibility of having giant intravesical prostate mimicking bladder carcinoma and the importance of cystoscopy in differentiating the two. Transvesical simple open prostatectomy has promising result.

## Highlights

• Benign prostate enlargement is a common cause of lower urinary tract symptoms among elderly men.
• Large intravesical prostatic protrusion is a rare condition, mimicking bladder tumour.
• Cystoscopy plays an important role in differentiating IPP from bladder tumour.
• Transvesical simple open prostatectomy for large intravesical prostatic protrusion and has good post operative results.

## 1 Introduction

Benign prostatic hyperplasia (BPH) is characterised histologically by increase number of prostatic epithelia and stromal cells. BPH is not uncommon condition among elderly men aged 50 years and above with prevalence that increases almost linearly and approaches 90 % at the age of 90 years [1], commonly presenting with enlargement of the lateral lobes. Globally, giant BPH of the median lobe is a rare occasion. It is usually characterised by overgrowth of the prostatic adenoma in the bladder lumen, a condition known as intravesical prostatic protrusion (IPP) [2]. Here in we present a case of a giant median lobe hyperplasia of the prostate posing diagnostic challenge.

## 2 Case presentation

A 71-year old male patient presented to our facility with complaint of draining urine per suprapubic catheter for 3 months following acute urine retention and a failure of passing urethral catheter. The urine retention was preceded by long standing history of lower urinary tract symptoms (LUTs) characterised by increased urinary frequency, weak stream, sense of incomplete bladder emptying and two episodes of painless gross haematuria. However he denied history of tobacco use or being exposed to chemical carcinogens or pelvic irradiation.

Upon general examination he was fully conscious, afebrile and pink eye sclera. He had a blood pressure of 132/84 mmHg, Pulse rate of 68 bpm, Temperature 36.7*C and respiratory rate of 17breaths/min. Per abdominal examination revealed a 22Fr suprapubic catheter connected to a urine bag that contained clear urine. The abdomen was of normal contours, no masses or organomegaly felt. Digital rectal examination revealed prostatomegaly with benign features. Other systems were essentially normal.

A diagnosis of bladder outlet obstruction (BOO) secondary to urethral stricture with a differential diagnosis of BOO secondary to prostate enlargement was entertained.

Laboratory worked up results is as shown on Table 1 below.

**Table 1:** Results for laboratory investigations.

sn	Name of the investigation	Results	Normal ranges
1	Prostate specific antigen	6.1 ng/mL	<4 ng/mL
2	Serum creatinine	75 μmol/L	62–106 μmol/L
3	Random blood sugar	5.24 mmol/L	<11.1 mmol/L
4	Full blood count		
	Hemoglobin level	11.7 g/dL	13–18
	Platelets	386 × 10^9^/L	150–500
	Leucocytes	5.22 × 10^9^/L	4–11
5	Urine culture	*Klebsiella oxytoca*	
	**Sensitivity**	Meropenem	
	**Resistance**	Ceftriaxone	
		Cefepime	
		Ciprofloxacin	
		Cefotaxime Gentamycin	
		Trimethoprim/sulfamethoxazole	
		Piperacillin/Tazobactam	

**Fig. 1:**
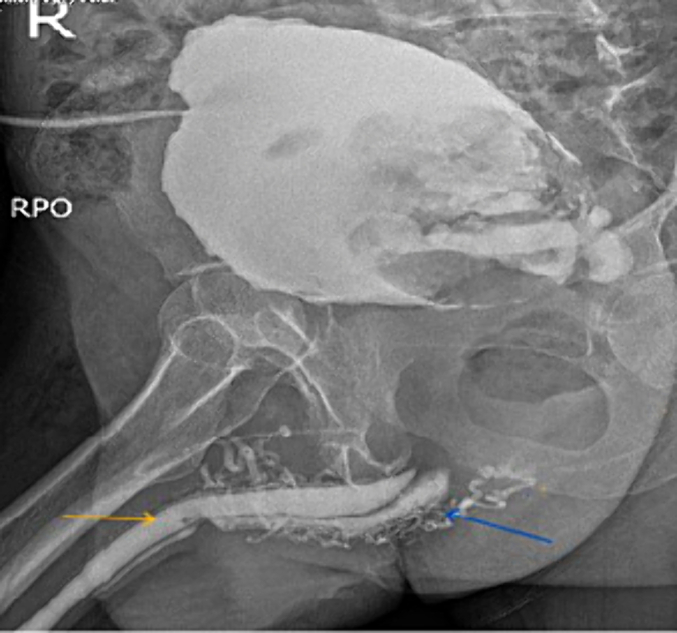
A simultaneous urethrogram showing normal anterior urethra with normal tapering of the bulbomembranous junction (yellow arrow), contrast extravasation (blue arrow) and a filling defect in the bladder.

Antegrade & retrograde urethrogram revealed normal anterior urethral with normal tapering at the bulbomembranous junction, contrast extravasation at the bulbous urethra and closed bladder neck with large filling defect in the urinary bladder (Fig. 1).

**Fig. 2:**
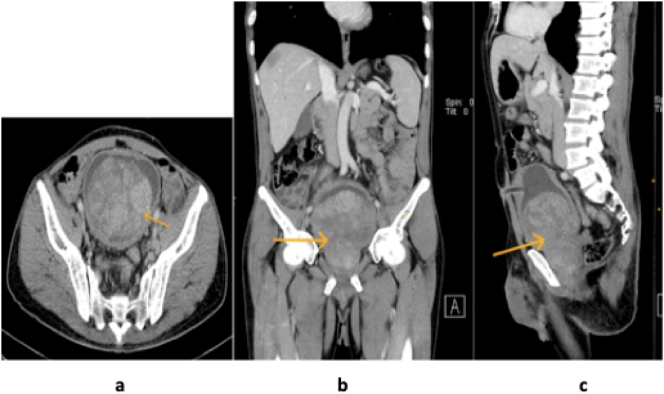
a, b & c: Showing a heterogeneous enhancing soft tissue mass (yellow arrows) arising from the posterolateral bladder wall, mass measured approximately 8.89 cm × 8.4 cm × 6.93 cm. (For interpretation of the references to colour in this figure legend, the reader is referred to the web version of this article.)

Ultrasound of the abdomen showed a well-defined heterogeneous mass in the urinary bladder measuring 8.6 cm × 11.1 cm with normal upper tracts. Due to this findings and the history of haematuria a Computer Tomography (CT) with Intravenous contrast was performed and showered; a heterogeneous enhancing soft tissue mass arising from the posterolateral bladder wall, measuring approximately 8.89 cm × 8.4 cm × 6.93 cm (Fig. 2a, b & c). The prostate measured 5.75 cm × 6.73 cm × 5.05 cm. However, there was no metastatic bone lesions noted or evidence of free fluid in the peritoneum. The pre and paravertebral soft tissues and other abdominal structures appeared normal.

He underwent examination under anaesthesia (EUA) and cystoscopy where, there was a smooth, mobile bladder mass measuring 5×7 cm was palpated. Using 16fr flexible scope, both anterior and membranous part of the urethral were normal, there was a giant median lobe of the prostate protruding into the bladder and occupying two third of the lumen. There were extensive trabeculations and sacculations in the bladder with obscuring of ureteric orifices by the giant median lobe. However, there were no urinary bladder diverticulum, stones or exophytic mass arising from its mucosae.

**Fig. 3:**
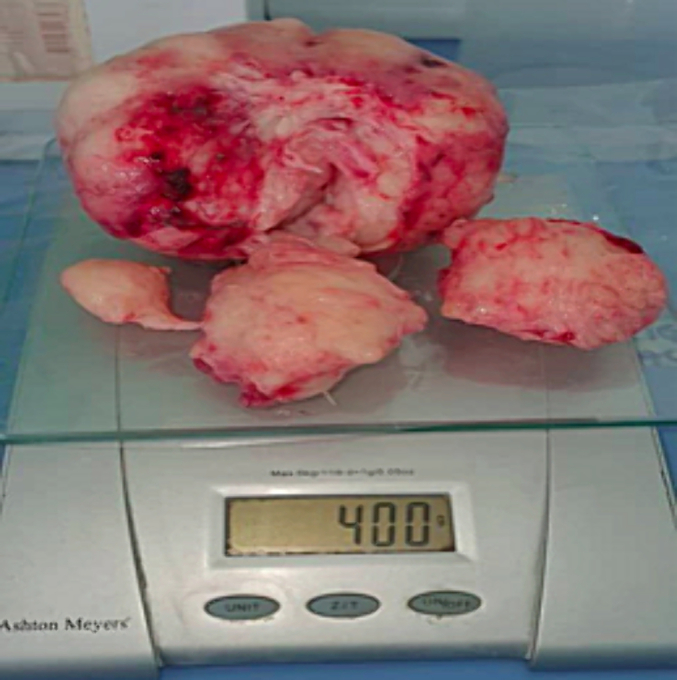
Showing the weight of the removed prostate placed on an Ashton Meyers® digital weighing machine.

**Fig. 4:**
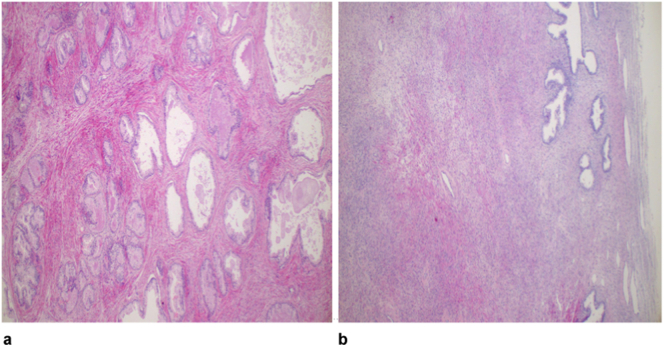
a: Histopathology showing a benign prostate nodular lesion with proliferation of stromal and glandular components, the latter is composed of variably sized glandular structures lined by basal and secretory cells, H&E staining 40× original. Fig. 4b: Photomicroscopy demonstrating stromal nodule composed of bland spindle cells with round to ovoid nuclei with open chromatin, H&E staining 40× original magnification.

He underwent simple transvesical open prostatectomy where a prostatic adenoma weighing 400 g was enucleated (Fig. 3). The surgery took a total of 2 h with no any intraoperative complications encountered. The histological analysis of the removed adenoma showed benign prostate nodular lesion with proliferation of stromal and glandular components, the latter was composed of variably sized glandular structures lined by basal and secretory cells. These findings were consistent with BPH (Fig. 4a, b). A 22fr 3 way urethral catheter inserted and continuous irrigation was initiated. Post operatively the patient was kept on continuous bladder irrigation with normal saline for 1 day, intravenous Meropenem for five days, intramusclular pethidine for 1-day then oral paracetamol and tramadol for 3 days. The abdominal tube drain was removed after 3 days and urethral catheter on day 10 post operative and allowed home. He was attended at the clinic three month later where he reported to be fine with normal voiding pattern. This case report has been reported in line with the SCARE 2023 criteria [3].

## 3 Discussion

IPP usually occurs on the median lobe of the prostate by growing along the less resistance plane, enter and occupy significant volume of bladder lumen and rarely mimic bladder carcinoma [2,4]. Like in our case, the enlarged prostate occupied large volume of the urinary bladder and it was confused with bladder carcinoma.

Both BPH and bladder tumour are prevalent in elderly men and they often exhibit lower urinary tract symptoms (LUTS) [4]. About 2.5 % of BPH cases present with gross haematuria [4], which is a common presenting symptoms for bladder carcinoma in >80 % of cases [5]. The cause of haematuria in BPH may be due to hypervasularity of the prostate with friable vessels that can be disrupted by strenuous physical activity [6]. Overlap of some of the risk factors and clinical presentation make it difficult to differentiate the two conditions clinically. Our patient was elderly in his 7th decade and presented with history of haematuria among others symptoms and we couldn't differentiate BPH from bladder carcinoma clinically. Since the two conditions, are treated differently, it is paramount important to differentiate them before initiating treatment.

Like in our case, both ultrasound and CT scan may all suggest bladder carcinoma [4,7]. Magnetic resonance imaging (MRI) and cystoscopy are useful investigation to differentiate IPP from primary bladder carcinoma [1,8]. We did not have facilities for MRI, however, cystoscopy was suggestive of giant IPP and therefore appropriate treatment was suggested.

IPP is classified/graded as grade 1, 2 and 3, which is equivalent to intravesical prostate length of <5 mm, 5–10 mm and >10 mm, respectively [1]. Our case had grade 3 IPP evidenced by the dimension of what was thought to be bladder carcinoma by the CT scan as well as abdominal ultrasound.

Although clear definition of giant median lobe does not exist in the literature, Senel et al. termed hyperplasia of median lobe of 180 g, giant [4]. Similarly, Ibrahim et al. termed median lobe of 225 g giant IPP [7]. In our case the IPP was giant as it weighted 400 g upon removal.

There are various treatment options for symptomatic BPH, including medical and surgical options. Medical treatment of giant IPP due to BPH with alpha-blockers and 5-alpha reductase inhibitors is ineffective in relieving the LUTS [9]. Hence, surgery remains the mainstay treatment with trans urethral resection of the prostate being the gold standard management of BPH [1] [9] [10]. However, for the prostate >80 g, open prostatectomy is the preferred surgical approach due to its less morbidity and mortality. There are two main surgical approaches for simple open prostatectomy, transvesical and retropubic techniques. The transvesical is the preferred technique in the presence of giant IPP, bladder diverticulum or bladder stone [4,11] due to easy accessibility and removal of the abnormality. Some patients might experience difficulties in micturition post operatively that eventually improve with time [1]. Similarly, in this case report a transvesical approach was used to enucleate the prostatic adenoma and upon removal of the catheter on day ten post operatively the patient reported to be passing urine normally where, he had low post void residual urine volume of 10mLs. The enucleated prostatic adenoma revealed findings suggestive of BPH.

## 4 Conclusion

IPP is rare and poses a diagnostic challenge that may delay treatment. Cystoscopy is a simple procedure that plays important role in arriving at the appropriate diagnosis. The treatment of choice for giant IPP is simple transvesical open prostatectomy, which has shown good prognosis in terms of alleviating the symptoms.

## Consent

Written informed consent was obtained from the patient for publication of this case report ad accompanying images. A copy of the written consent is available for review by the editor-in-chief of this journal on request.

## Provenance and peer review

Not commissioned, externally peer reviewed.

## Ethical approval

The author's institution review board waived ethical approval.

## Funding

There work has not received any funds from individual or organization.

## CRediT authorship contribution statement

BNN, MK, FB were involved in the surgical procedure. MK extracted patient information from case noted. BNN, MK managed the patient post operatively. AM interpreted the histological slide and prepared the images. OJM, JL, JM, AM reviewed the initial draft.

## Guarantor

Bartholomeo Nicholaus Ngowi is the guarantor of this work.

## Declaration of competing interest

All listed authors have not conflict of interest to declare.
